# Effect of Synthesis Temperature on Structure and Magnetic Properties of (La,Nd)_0.7_Sr_0.3_MnO_3_ Nanoparticles

**DOI:** 10.1186/s11671-017-1884-4

**Published:** 2017-02-08

**Authors:** Yulia Shlapa, Sergii Solopan, Andrii Bodnaruk, Mykola Kulyk, Viktor Kalita, Yulia Tykhonenko-Polishchuk, Alexandr Tovstolytkin, Anatolii Belous

**Affiliations:** 1 V.I. Vernadskii Institute of General and Inorganic Chemistry of the NAS of Ukraine, 32/34 Palladina Ave., Kyiv, 03142 Ukraine; 2 Institute of Physics of the NAS of Ukraine, 46 Nauky Ave., Kyiv, 03028 Ukraine; 3 Institute of Magnetism of the NAS of Ukraine and MES of Ukraine, 36-b Vernadsky Blvd., Kyiv, 03680 Ukraine

**Keywords:** Manganite nanoparticles: magnetic hyperthermia, Distorted perovskite structure, Oxygen non-stoichiometry, Magnetization, Curie point, Coercivity, Specific loss power (SLP), 61.46.Df, 75.75.Cd, 81.07. Bc

## Abstract

Two sets of Nd-doped La_0.7_Sr_0.3_MnO_3_ nanoparticles were synthesized via sol-gel method with further heat treatment at 1073 and 1573 K, respectively. Crystallographic and magnetic properties of obtained nanoparticles were studied, and the effect of synthesis conditions on these properties was investigated. According to X-ray data, all particles crystallized in the distorted perovskite structure. Magnetic parameters, such as saturation magnetization, coercivity, Curie temperature, and specific loss power, which is released on the exposure of an ensemble of nanoparticles to AC magnetic field, were determined for both sets of samples. The correlation between the values of Curie temperature and maximal heating temperature under AC magnetic field was found. It was revealed that for the samples synthesized at 1573 K, the dependences of crystallographic and magnetic parameters on Nd content were monotonous, while for the samples synthesized at 1073 K, they were non-monotonous. It was concluded that Nd-doped La_0.7_Sr_0.3_MnO_3_ nanoparticles are promising materials for self-controlled magnetic hyperthermia applications, but the researchers should be aware of the unusual behavior of the particles synthesized at relatively low temperatures.

## Background

Nanoparticles of ferro- and ferrimagnetic materials have been of particular scientific and practical interest due to their possibility to be applied in medicine [1–4] The most promising fields of medical applications are targeted drug and bioactive object delivery [5, 6], cellular monitoring of bioactive systems (for example, magnetic resonance imaging (MRI)) [7], magnetic hyperthermia [8, 9], etc. The close relation between modern development of cellular and tissue technology and the latest developments in biochemistry, biophysics, and nanotechnology has been at the heart of such interest [10, 11].

At present, researchers consider spinel nanoferrites (for instance, magnetite Fe_3_O_4_) as principal candidates for various applications in biomedicine. Iron oxide nanoparticles have already been used as the contrast agents in MRI. They significantly enhance MRI signal and cause minimal side effects on living organisms [12]. At the same time, new materials are examined with the aim to improve efficiency and widen the areas of applications. In vivo studies [13] show that MnFe_2_O_4_ nanoparticles display much better characteristics than Fe_3_O_4_ ones, while using them as contrast agents for MRI. The advantage of Tb-doped Fe_3_O_4_ nanoparticles consists not only in enhanced magnetic characteristics but also in the possibility to investigate the fluorescent properties [14]. It should be mentioned that all these materials did not demonstrate any cytotoxicity during cytological studies [15].

A great deal of work has been aimed at a search for new materials for magnetic hyperthermia applications [9, 10, 16]. To be successfully used in hyperthermia therapy, magnetic nanoparticles have to meet some important requirements, such as to be biocompatible, weakly agglomerated (with the aim to prevent the clots formation), and characterized by the absence of residual magnetization. In addition, they have to heat up effectively under the action of an AC magnetic field [16]. However, the heating of tumor tissue without damaging the surrounding structures has been a challenge to scientists. In the presence of an AC magnetic field, the heat produced by the particles cannot be controlled, as these particles retain their magnetic properties even at sufficiently high temperatures. The temperature at which they change their magnetic behavior from ferro- or ferrimagnetic to paramagnetic is called Curie temperature (*T*_*C*_). Thus, *T*_*C*_ point near 50 °C can be used as in vivo switch, since in the presence of AC magnetic field, the temperature will not rise above it, and further heating will stop [17].

The nanoparticles of spinel ferrites display relatively high values of *T*_*C*_ (for instance, *T*_*C*_ of Fe_3_O_4_ is 585 °C) [18]. At the same time, the materials like La_1−*y*_Sr_*y*_MnO_3_ look very promising for the hyperthermia applications, since they have relatively large magnetic moment at room temperature and their phase-transition temperature can be easily tuned in the range of 0–90 °C by changing the chemical composition [19].

Lanthanum-strontium manganites La_1−*y*_Sr_*y*_MnO_3_ crystallize in the distorted perovskite structure and display ferromagnetic properties over the wide range of Sr-content (0.15 ≤ *y* ≤ 0.60) [20, 21]. Their Curie temperature strongly depends on the chemical composition: it demonstrates the maximal value at *y* ≈ 0.3 (*T*_*C*max_ ≈ 370 K) and quite sharply decreases as *y* deviates from 0.3. Earlier, it was shown that fine controlling of the Curie point within the narrow temperature range can be reached by low-level substitutions in manganese sublattice [22]. At the same time, partial substitutions of La^3+^ ions by other rare-earth ions (for instance, by Nd^3+^) may be one more possible way of fine-tuning the phase-transition temperature, since magnetic interactions in manganites are sensitive to the lattice parameters and strength of local lattice distortions [23]. Since the ionic radii of La^3+^ (1.36 Å) and Nd^3+^ (1.27 Å) ions are quite close, the Curie temperature is expected to be changed more softly upon substitutions of such kind.

Unfortunately, the properties of substituted manganites are very sensitive to the synthesis conditions. As shown in [21, 24–27], the changes of heating temperature or annealing duration may lead to the deviation of an oxygen content from stoichiometric value. According to [21], the stoichiometry of substituted manganites and oxidation state of Mn ions depend not only on Sr-content but also on atmosphere, temperature, and duration of heat treatment. Moreover, the results reported in [24, 26] indicate that the synthesis under the same conditions does not guarantee the same degree of oxygen non-stoichiometry in the samples with different chemical composition. The authors of [24] came to a conclusion that these effects are likely to originate from close interdependence between crystallographic parameters and oxygen diffusion coeficient: the contraction of crystal lattice makes oxygen diffusion more difficult, and this eventually results in the dependence of the degree of oxygen non-stoichiometry on such factors as structure, chemical composition, and others.

Up to now, the most of studies have been dealing with the substituted manganites obtained at relatively high temperatures (≥1300 K) [28, 29]. Recently, the investigation of manganite nanoparticles and their properties has become more relevant [30]. In most cases, lower temperatures are used in the process of the synthesis of nanoparticles (around 1000 K and lower) [31, 32]. Previous data show that in the latter case, the dependence of oxygen non-stoichiometry on the chemical composition can be quite significant (even for the synthesis of a series of samples under the same conditions) [21]. However, there are no any more detailed investigations of this issue.

The aim of this study is synthesis of La_0.7−*x*_Nd_*x*_Sr_0.3_MnO_3_ (*x* = 0–0.1) nanoparticles via sol-gel method with further annealing at different temperatures, investigation of their structural and magnetic properties, and clarifying the effect of the chemical composition and synthesis conditions on the crystallographic and magnetic parameters.

## Methods

Nd-doped (≤10%) manganite nanoparticles were synthesized via sol-gel method. The aqueous solutions of metal salts La(NO_3_)_3_, Nd(NO_3_)_3_, Sr(NO_3_)_3_, and Mn(NO_3_)_2_ were used as the starting reagents. Necessary amounts of starting solutions were mixed, citric acid and ethylene glycol were added as the gel forming agents. The obtained reaction mixture was heated with stirring at 353 K. The polymer gel was formed during polyesterification reaction, which took place at pH = 9. An amorphous precursor (La,Nd,Sr)MnO_3_, obtained after gel pyrolysis, was subjected to further high-temperature treatment for 2 h: one set at 1073 K, while another set at 1573 K.

X-ray diffraction study (XRD) of the obtained powders was performed using DRON-4 diffractometer (CuKα radiation). Crystallographic lattice parameters of the single-phased product were calculated by Rietveld method using FULL-PROF software package. The crystallite size was estimated from XRD line broadening using Scherrer equation:

1 $$ {D}_{\mathrm{XRD}}=\frac{K\cdot \lambda}{B\cdot \cos \theta} $$

where *λ* is a wave length, *B* is the peak width, *θ* is the Bragg angle, and *K* ≈ 0.89 is the shape factor [33].

Magnetic measurements were performed using a LDJ-9500 vibrating sample magnetometer. To analyze the field and temperature dependences of magnetization, hysteresis loops were measured for −5 kOe ≤ *H* ≤ 5 kOe in the temperature range from 110 to 370 K.

For the calorimetric determination of specific loss power (SLP) which is released on the exposure of an ensemble of the particles to AC magnetic field, the ferrofluids based on synthesized magnetic nanoparticles (50 mg/mL) were prepared using 0.1% aqueous agarose solutions. To obtain the fluid temperature *T*_fluid_ vs residence time *τ* dependences, the magnetic fluids were placed into the coil, which generated AC magnetic field with a frequency of 300 kHz and amplitude up to 9.5 kA/m. All measurements and calculations were done according to the procedure described in [34]. Specific loss power values were calculated by the formula:

2 $$ \mathrm{S}\mathrm{L}\mathrm{P}=\frac{C_{\mathrm{fluid}}\times {V}_s}{m_{\mathrm{powder}}}\times \frac{\mathrm{d}{T}_{\mathrm{fluid}}}{\mathrm{d}\tau} $$

where d*T*_fluid_/d*τ* is an initial slope of the temperature vs time dependence, *C*_fluid_ and *V*_*s*_ are the volumetric specific heat and the sample volume, respectively, and *m*_powder_ is the mass of the magnetic material in the fluid.

## Results and Discussion

**Fig. 1:**
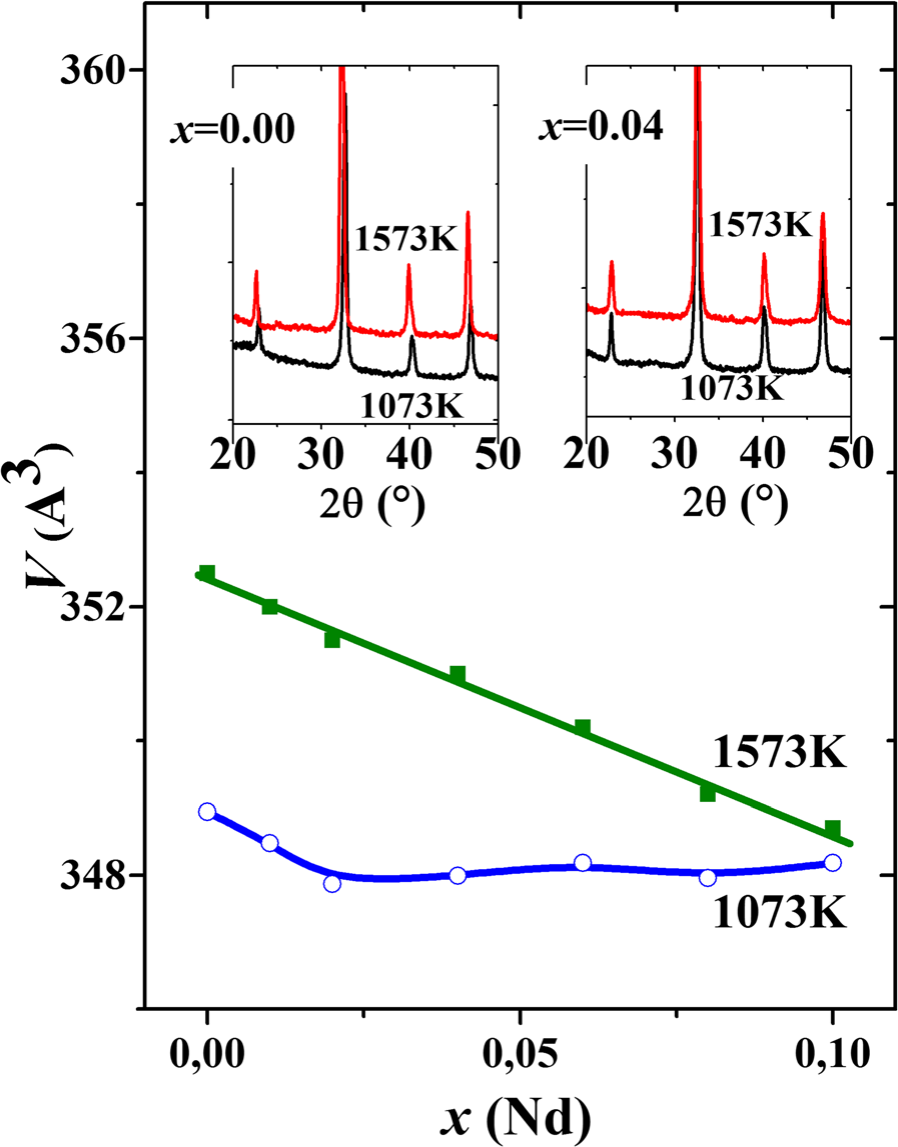
The unit cell volume vs Nd content for La_0.7−*x*_Nd_*x*_Sr_0.3_MnO_3_ nanoparticles synthesized at 1073 and 1573 K. Representative XRD patterns for nanoparticles with *x* = 0.00 and 0.04 are shown in the *insets*

According to XRD data, the synthesized La_0.7−*x*_Nd_*x*_Sr_0.3_MnO_3_ nanoparticles are single-phased and crystallize in the distorted perovskite structure (space group R-3c) even at 1073 K. Representative diffraction patterns for the samples with *x* = 0.00 and 0.04 are shown in the insets to Fig. 1. Corresponding structural parameters (*a = b, c, V)* for the nanoparticles, synthesized at 1073 K and 1573 K, were calculated by Rietveld method. The results are summarized in Table 1.

**Table 1:** Crystallographic parameters of La_0.7−*x*_Nd_*x*_Sr_0.3_MnO_3_ nanoparticles obtained via sol-gel method

*x*	0.00	0.01	0.02	0.04	0.06	0.08	0.1
La_0.7−*x*_Nd_*x*_Sr_0.3_MnO_3_ after 1073 K							
*a*, Å	5.4911(8)	5.4901(7)	5.4884(4)	5.4867(2)	5.4914(2)	5.4908(2)	5.4933(5)
*c*, Å	13.3632(1)	13.3498(2)	13.3351(1)	13.3482(7)	13.3326(7)	13.3270(9)	13.3232(1)
*V*, Å^3^	348.94(5)	348.48(9)	347.87(5)	347.99(2)	348.18(2)	347.96(3)	348.18(7)
*Rb*,%	5.60	9.14	7.06	5.07	4.52	5.87	8.24
*Rf*, %	8.19	12.8	9.35	6.36	5.77	7.57	11.5
*d*_XRD*,*_ *nm*	31	30	32	37	36	36	39
La_0.7−*x*_Nd_*x*_Sr_0.3_MnO_3_ after 1573 K							
*a*, Å	5.485(1)	5.482(1)	5.479(3)	5.476(2)	5.472(2)	5.466(1)	5.465(2)
*c*, Å	13.531(5)	13.524(2)	13.520(8)	13.517(6)	13.504(5)	13.498(6)	13.486(1)
*V*, Å^3^	352.5(2)	352.0(1)	351.5(3)	351.0(2)	350.2(2)	349.2(2)	348.7(4)
*Rb*, %	6.60	7.14	5.80	6.07	5.42	4.92	8.30
*Rf*, %	8.30	8.80	7.35	6.46	6.77	6.67	5.42

Here, *R*_*b*_ is Bragg factor and *R*_*f*_ is compliance form factor

As seen from the data of Table 1, the unit cell volume for La_0.7−*x*_Nd_*x*_Sr_0.3_MnO_3_ nanoparticles, synthesized at 1573 K, decreases with growing Nd content. Such behavior points toward isomorphic substitution of larger La^3+^ ions by smaller Nd^3+^ ions. It follows from Fig. 1 that there is a linear change of crystallographic parameters over the range of Nd from 0.0 to 0.1, which means that the system obeys Vegard’s law. Thus, a continuous line of solid solutions is formed in this Nd-concentration range. At the same time, the linear *V*(*x*) dependence implies that oxygen amount is the same (or quite close) in all samples and it is independent of the Nd concentration.

The non-monotonous relation between crystallographic parameters and Nd-content is observed for La_0.7−*x*_Nd_*x*_Sr_0.3_MnO_3_ nanoparticles synthesized at 1073 K (see Fig. 1). This fact can be caused either by incomplete ordering of the crystal structure or by the dependence of oxygen non-stoichiometry on the samples’ chemical composition (in this case—on the Nd content). Similar effects, but for the perovskite compounds of other compositions, were reported in [21, 24, 25, 35] and explained by the dependence of oxygen diffusion coefficient on lattice parameters [24] or by stress-induced clustering of oxygen vacancies [25]. It was also shown that Nd-containing manganites usually show a very varied defect structure: depending on composition and heat treatment, vacancies can form on any one or any two of the three sublattices (Ln, Mn or oxygen) [35]. To distinguish between different models of defect formation and mechanisms of stress accommodation in Nd-containing manganites, a number of additional studies and calculations are necessary, but such activities go beyond the scope of this work.

The crystallite sizes of nanoparticles obtained at 1073 K were calculated from the Sherrer’s formula with the use of (110) peak (Table 1). According to the calculations, the crystallite sizes are in the range of 30–39 nm.

**Fig. 2:**
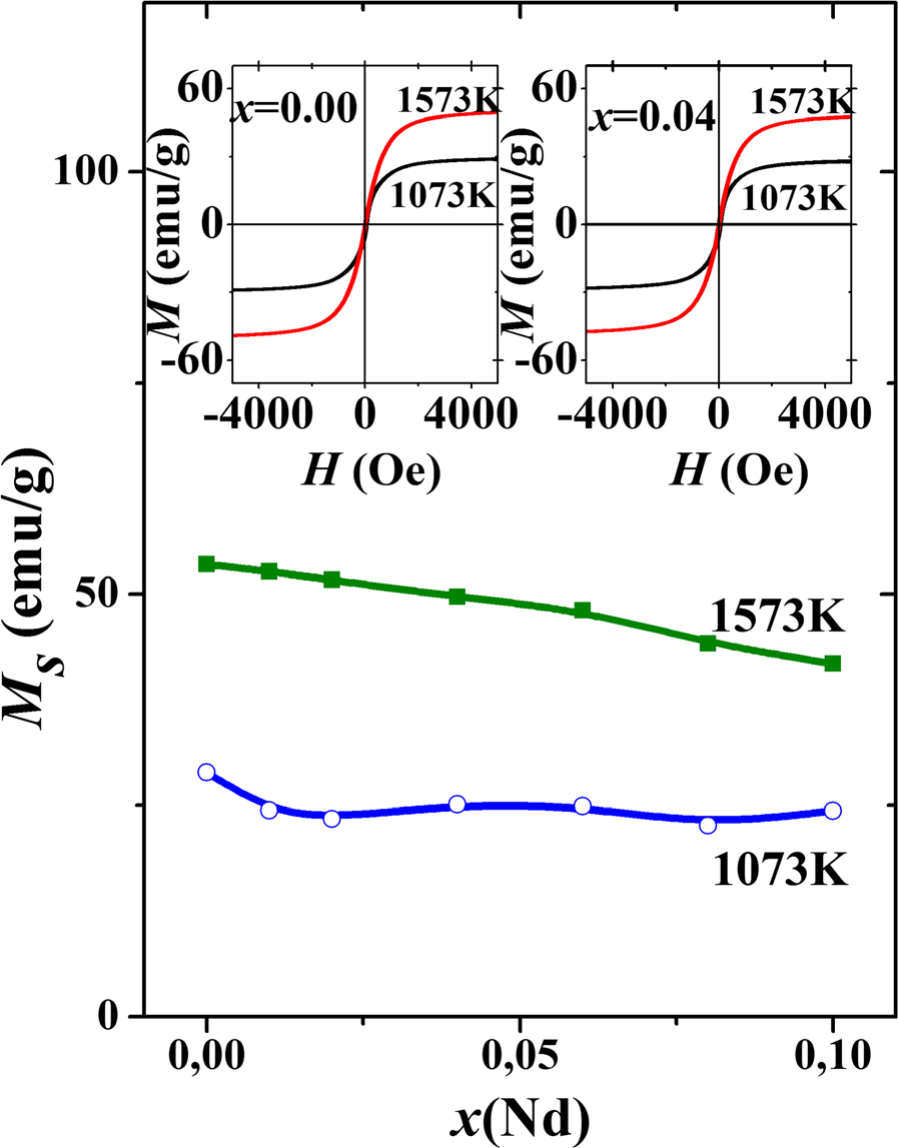
Saturation magnetization *M*_*s*_ vs Nd content for La_0.7−*x*_Nd_*x*_Sr_0.3_MnO_3_ nanoparticles synthesized at 1073 and 1573 K. Representative field dependences of the magnetization *M*(*H*) for nanoparticles with *x* = 0.00 and 0.04 are shown in the *insets*

Magnetization curves *M*(*H*) for La_0.7−*x*_Nd_*x*_Sr_0.3_MnO_3_ nanoparticles with *x* = 0.00 and 0.04, synthesized at different temperatures, are shown in the insets to Fig. 2. The measurements were performed at 295 K. Magnetization grows with increasing magnetic field and tends to saturation at *H* ≥ 1500 Oe. The general trend is the reduction of saturation magnetization *M*_*s*_ with the increase in Nd content. Also, *M*_*s*_ becomes almost two times lower as synthesis temperature decreases from 1573 to 1073 K.

Figure 2 presents the concentration dependence of the saturation magnetization, *M*_*s*_(*x*), for the samples synthesized at 1573 and 1073 K. In the first case, *M*_*s*_ is the monotonous and almost linear function of *x*. What concerns the second case (synthesis temperature is 1073 K), the saturation magnetization is almost twice lower than *M*_*s*_ for the first series and its values are only weakly dependent on *x*. The character of the variation of *M*_*s*_(*x*) for the samples synthesized at lower temperature is close to that of *V*(*x*) dependence (see Fig. 1), which indicates that the peculiar features of both dependences may be of the same nature.

**Fig. 3:**
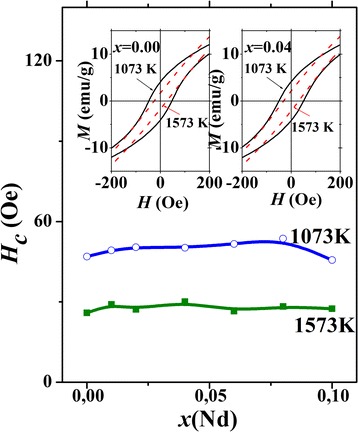
Concentration dependences of coercivity *H*_*c*_ for La_0.7−*x*_Nd_*x*_Sr_0.3_MnO_3_ nanoparticles synthesized at 1073 and 1573 K. The *insets* show low-field regions of representative *M*(*H*) dependences for nanoparticles with *x* = 0.00 and 0.04

More details of the magnetization behavior can be seen from Fig. 3, where *M*(*H*) dependences in the range of the weak magnetic fields are shown. It follows from the *M*(*H*) data that the coercivity *H*_*c*_ for the nanoparticles synthesized at lower temperatures (1073 K) are 1.5–2 times higher than *H*_*c*_ for the nanoparticles synthesized at higher temperature. According to Fig. 3, where the *H*_*c*_(*x*) dependences for both series of the samples are shown, the coercivity in both cases is weakly dependent on the Nd content. Strong increase in the coercivity, observed in the first series of samples, is likely to originate from the enhanced inhomogeneity of these samples.

Magnetic state of nanoparticles is usually quite inhomogeneous due to significant contribution from the surface layer, whose properties differ from the properties of the nanoparticle core, and due to the scatter in particle size. It is shown in [36, 37] that the temperature behavior of particle ensembles can be satisfactory as described by the average Curie temperature concept. In this case, it is expected that the behavior of the ensembles’ magnetization will obey the law *M(T)* $\sim \sqrt{T_C- T}$.

**Fig. 4:**
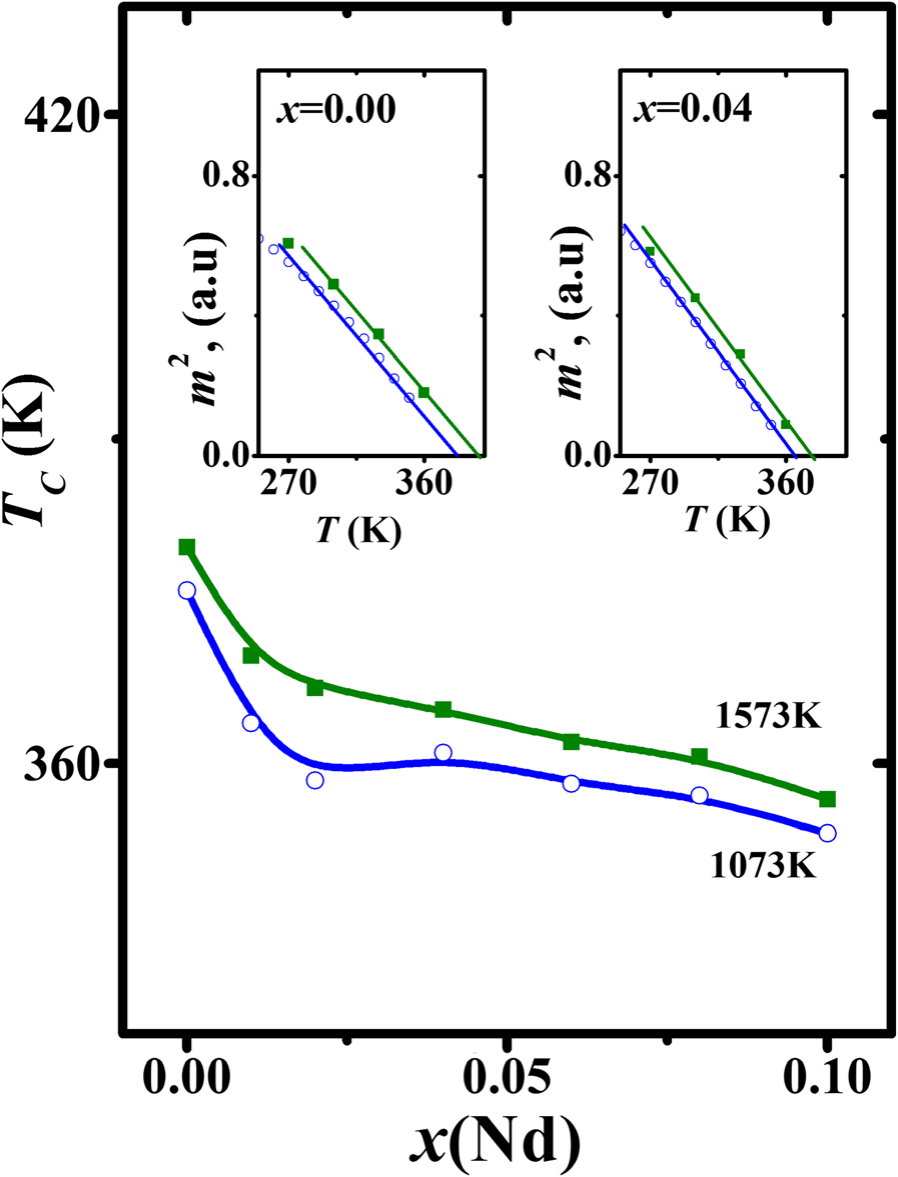
Concentration dependences of *T*_*C*_ for La_0.7−*x*_Nd_*x*_Sr_0.3_MnO_3_ nanoparticles synthesized at 1073 and 1573 K. The *insets* show representative temperature dependences of the square of normalized magnetization for nanoparticles with *x* = 0.00 and 0.04

The insets to Fig. 4 show the representative temperature dependences of the square of normalized magnetization *m* = *M*(*T*, *H* = 5 kOe)/*M*(110 K, *H* = 5 kOe) for La_0.7−*x*_Nd_*x*_Sr_0.3_MnO_3_ nanoparticles synthesized at 1573 and 1073 K. It is seen that there is a wide temperature range where such dependences are linear (see straight solid lines in the insets to Fig. 4). The Curie temperature values can be estimated from these dependences by the points of intersection of linear area with the temperature axis. Concentration dependences of *T*_*C*_(*x*) for both series of samples are shown in Fig. 4. In both cases, *T*_*C*_ decreases with growing Nd content. At the same time, all samples synthesized at lower temperature have lower Curie point.

To characterize the heating efficiency of the nanoparticles, magnetic fluids based on them and aqueous agarose solution were prepared and subjected to AC magnetic field. Dependence of the temperature of magnetic fluid (*T*_fluid_) vs time resided in AC field (*τ*) with fixed amplitude *H*_max_, and frequency *f* makes it possible to calculate SLP values.

**Fig. 5:**
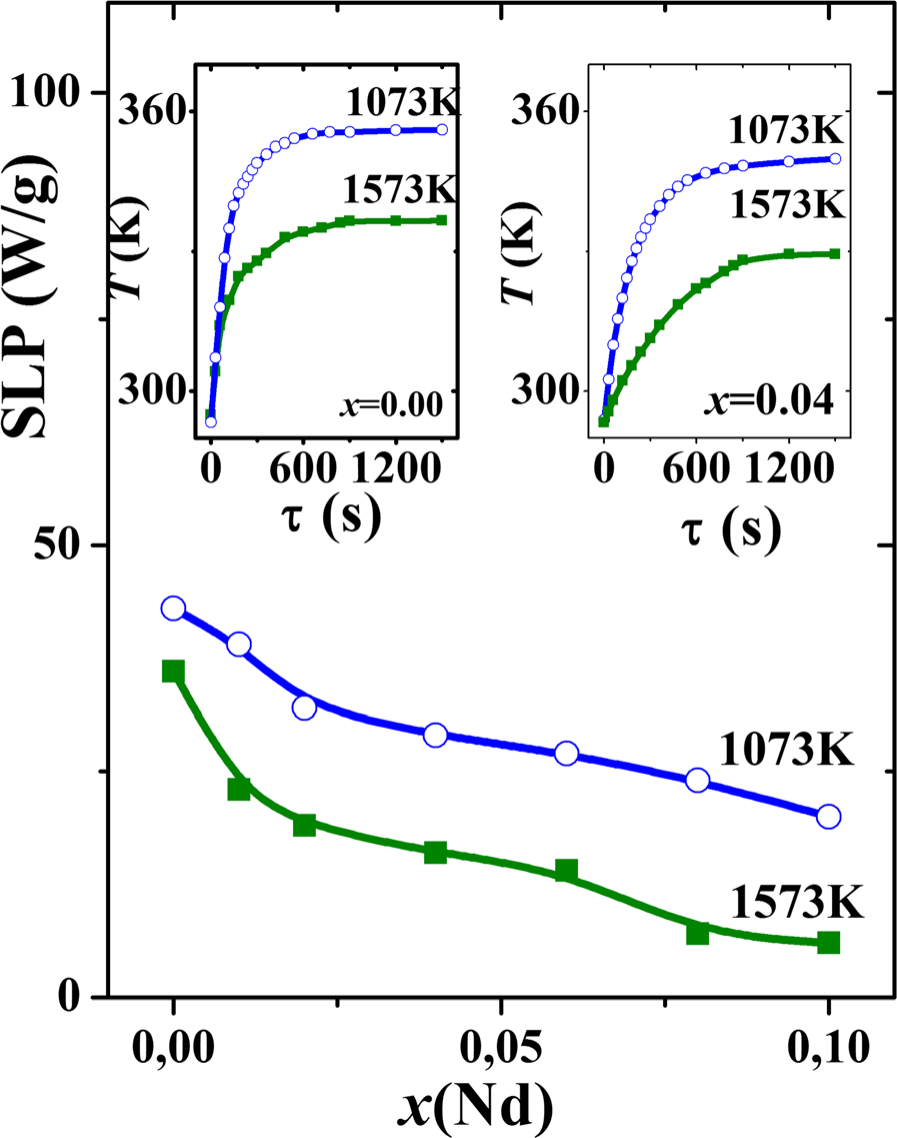
Concentration dependences of SLP values for La_0.7−*x*_Nd_*x*_Sr_0.3_MnO_3_ nanoparticles synthesized at 1073 and 1573 K. The *insets* show representative *T*_fluid_ vs *τ* dependences for magnetic fluids based on nanoparticles with *x* = 0.00 and 0.04

Representative *T*_fluid_(*τ*) dependences for magnetic fluids based on La_0.7−*x*_Nd_*x*_Sr_0.3_MnO_3_ nanoparticles with *x* = 0.00 and 0.04 are shown in the insets to Fig. 5. The measurements were performed in magnetic field with amplitude *H*_max_ = 9.3 kA/m and frequency *f* = 300 kHz. It can be seen that all samples effectively heat up under the action of AC magnetic field. However, the heating efficiency of manganites decreases with growing the Nd content.

Figure 5 shows the SLP values calculated according to formula (2). It should be mentioned that the application of these nanoparticles allows one to provide heating to certain constant temperature (saturation temperature *T*_*S*_). An interesting feature is that SLP values for nanoparticles synthesized at higher temperature are lower, than corresponding values for those synthesized at lower temperature. The insets to Fig. 3 may provide the explanation for this fact: the coercivity of the samples synthesized at 1073 K exceeds *H*_*c*_ for the samples obtained at 1573 K. At the same time, the magnetization in the weak-field range does not strongly differ for both kinds of samples. As a result, the area of the hysteresis loop in the first case is greater than in the second case. Since SLP is proportional to the area of hysteresis loop [37], its values are greater in the samples synthesized at 1073 K.

**Fig. 6:**
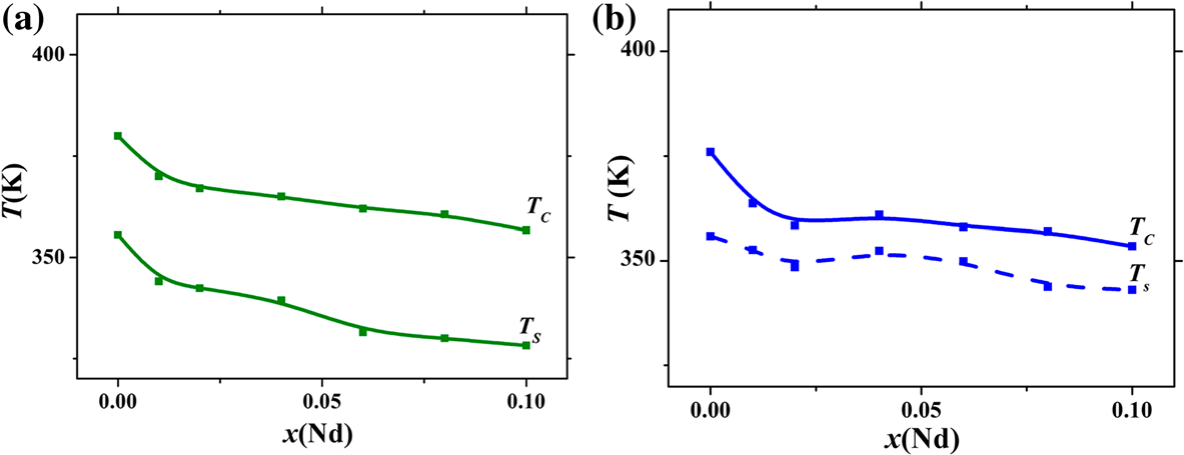
Concentration dependences of the *T*_*C*_ and *T*_*s*_ values for La_0.7−*x*_Nd_*x*_Sr_0.3_MnO_3_ nanoparticles synthesized at 1573 (**a**) and 1073 K (**b**)

It is seen from the insets to Fig. 5 that after sharp initial rise, the change of *T*_fluid_ with *τ* becomes slower and eventually *T*_fluid_ goes to saturation. The saturation temperature *T*_*s*_ decreases with the increase in the Nd content. Figure 6 compares the *T*_*C*_ and *T*_*s*_ values for La_0.7−*x*_Nd_*x*_Sr_0.3_MnO_3_ nanoparticles synthesized at different temperatures. It is seen that the character of *T*_*C*_ vs *x* dependences is close to that of *T*_*s*_ vs *x* ones. At the same time, the values of *T*_*s*_, although being close to *T*_*C*_, never exceed the *T*_*C*_ values. This implies that heat transfer to the surrounding environment may play an important role, and such effect should be taken into account when using the magnetic fluids under real conditions.

Based on the results of complex investigations, one can conclude that the modification of La_1−*y*_Sr_*y*_MnO_3_ manganite nanoparticles, particularly by partial substitution of La by Nd, strongly affects their properties. At the same time, crystallographic and magnetic parameters may display non-monotonous dependence on Nd content, and this phenomenon may become more pronounced in the samples synthesized at lower temperatures.

## Conclusions

The effect of synthesis temperature on the structural and magnetic properties of La_0.7−*x*_Nd_*x*_Sr_0.3_MnO_3_ (*x* = 0.00–0.1) nanoparticles was investigated in this study. It was shown that for nanoparticles synthesized at 1573 K, both crystallographic and magnetic parameters monotonously change with changing Nd content. The obtained result are in good agreement with literature data for bulk counterparts and can be explained by the enhancement of the local distortions of crystal lattice upon partial substitution of La^3+^ ions by Nd^3+^ ones.

For the samples synthesized at 1073 K, both crystallographic and magnetic parameters display non-monotonous dependence on Nd content. This fact can be explained either by incomplete ordering of the crystal structure or by the dependence of the degree of oxygen non-stoichiometry on chemical composition (in this case—on Nd content). Since magnetic nanoparticles are mainly synthesized at relatively low temperatures, such features have to be taken into account in researching and predicting the particles’ properties.
